# Supplementary material for: Gaze-Contingent Eye-Tracking Training in Brain Disorders: A Systematic Review
Source: Brain Sci. 2022 Jul 16;12(7):931. doi: 10.3390/brainsci12070931 (PMC9313363; doi:10.3390/brainsci12070931)
Supplement: Supplementary file 1 [file brainsci-12-00931-s001.zip › Table S1.pdf]

**PUBMED**
((Eye Tracking[Title/Abstract]) OR (Eye-Tracking[Title/Abstract]) OR (Oculomotor[Title/Abstract])) AND ((Neuropsychol*[Title/Abstract]) OR (Cognit*[Title/Abstract])) AND ((Training[Title/Abstract]) OR (Rehabilitation[Title/Abstract]) OR (Stimulation[Title/abstract]))
**SCOPUS**
TITLE-ABS (("Eye Tracking" OR "Eye-Tracking" OR "Oculomotor") AND ("neuropsychol*" OR "cognitive") AND ("rehabilitation" OR "training" OR "stimulation")) AND (LIMIT-TO (PUBSTAGE , "final")) AND (LIMIT-TO (LANGUAGE , "English"))
**Web Of Science**
AB=(((Eye AND Tracking) OR (Eye-Tracking) OR (Oculomotor)) AND ((Neuropsychol*) OR (Cognitive)) AND (Training OR Rehabilitation OR Stimulation))
