# Supplementary material for: Gaze-Contingent Eye-Tracking Training in Brain Disorders: A Systematic Review
Source: Brain Sci. 2022 Jul 16;12(7):931. doi: 10.3390/brainsci12070931 (PMC9313363; doi:10.3390/brainsci12070931)
Supplement: Supplementary file 1 [file brainsci-12-00931-s001.zip › Table S2.pdf]

Article	Sample Information	Main Findings	ET Device (Company)	LoE
Psychiatric diagnosis				
Ferrari GRA et al, 2016	Students in Netherland. Mean age: 21.79 y	The PT (training to disengage attention from negative pictures and shift it towards positive ones) induced longer fixations on positive pictures and faster disengagement from negative pictures. The NT (opposite pattern) showed no changes in attentional processes.	iView 9 Hi-Speed (SMI)	II
Kvamme TL et al, 2019	Patients with alcohol dependence (AUDIT > 20). Mean age: 48.9 y	ABM coupled with ET-based operant conditioning of gaze behavior reduces AB on trained images within the trained target duration. No significant effects found on other AB-components, nor on craving to specific alcohol images or addiction symptomology both following the intervention and at 3-month follow-up.	Tobii X2-60 (Tobii)	II
Lazarov A et al, 2016	Patients with social anxiety disorder (SPIN ≥30). Mean age 33.83 y.	Gaze-contingent music reward therapy yielded greater reductions of symptoms of social anxiety disorder than the control condition, maintained at follow-up. Gaze-contingent music reward therapy, but not the control condition, also reduced dwell time on threat and altered dwell time on socially threatening faces not used in training.	SMI RED500 (iMotions)	II
Price I et al, 2016	Healthy undergraduate students. Mean age: 20.56 y	Operant conditioning of eye gaze towards happy faces buffers against stress-induced effects on mood, particularly in individuals who show sufficient baseline neural engagement with happy faces.	EyeLink II (SR research)	II
Sanchez-Lopez A et al, 2019	Undergraduate students. Mean age 23.30 y	Active ECAT resulted in more sustained attention on positive information and larger reductions in state rumination after viewing negative scenes.	Tobii TX-300 (Tobii)	II
Vazquez C et al, 2016	19-30 y dysphoric university students (BDI - II > 13 points)	NA	Tobii TX-120 (Tobii)	NA
Woolridge SM et al, 2020	18-65 y with diagnosis of major depressive disorder	Training reduced negative attentional biases. Relative to sham, active training participants focused significantly more on positive compared to negative stimuli in a free-viewing ET task and, at trend, disengaged from sad information more quickly in a computerized task; remembered more happy than sad words in an emotional word learning task. No effects on mood in the one-week trial.	NA	II

Neurodevelopmental conditions				
Ballieux H et al, 2016	Typically developing infants, age range 11 M 0 D-12 M 30 D	Training-related improvements were found, relative to active controls, on tasks assessing visual sustained attention, saccadic reaction time, and rule learning, whereas trend improvements were found on measures of short-term memory. No significant improvements were found in task-switching.	Tobii T120 (Tobii)	II
Dovigo L et al, 2021	Females with RS. Mean age: 9.8 y	Participants attended both social and cognitive tasks with spontaneous reduction of stereotypes and with increased attention. They recalled more significant indexes when music or songs were presented within the cartoon or the cognitive task.	NA	VI
Fabio RA et al, 2018	Patients with RS, aged from 2 to 33 y (mean: 12.10 y)	Performance enhancement in attention and motivation.	Tobii Series-I (Tobii)	II
García-Baos, A et al, 2019	Children aged 8-15 y with ADHD	Participants in the ET group showed a post-test improvement compared to pre-test in impulsivity, reaction times, and fixation gaze control. No changes were found in mouse control between pre-test and post-test measurements.	Tobii EyeX (Tobii)	II
Garcia-Zapirain B et al, 2017	HCs, mean age 10.88 y	The solution proposed in order to help children with attention and learning problem was an entertaining alternative to both hand-writing and the use of the keyboard and mouse, which were found as more boring and consequently distractive.	Tobii X1 Light (Tobii)	VI
Goodwin A et al, 2016	10–14 M infants with a first-degree relative with ADHD	NA	Tobii X2-60 (Tobii)	NA
Iannizzotto G et al, 2020	Females with RS, in their late primary and secondary school ages	Preliminary results: the correspondence between the results of the SWYG eye tracker and the Tobii ET was ≈98%.	Tobii 4C (Tobii)	VI
Lee TL et al, 2021	ADHD patients. Age range: 6-12 y	The experimental group exhibited significant improvements in tests of inhibition (Flanker test, Category Fluency and Five-Point Tests, Trail 2 of the Children’s Color Trail Test). The control group did not show significant changes.	Tobii 4C (Tobii)	II

Lee TL et al, 2020	ADHD patients. IQ > 70. Age range: 6-12 y	In the experimental group, significant improvements in saccade latency and accuracy in the anti- and pro-saccade tasks were observed, respectively.	Tobii 4C (Tobii)	II
Perra O et al, 2020	Healthy very preterm infants at 12 months of age	NA	NA	NA
Perra O et al, 2021	Healthy very preterm infants at 12 months of age	The rate of completion of baseline and outcome measures was optimal. VP infants demonstrated engagement in the training, completing on average 84 min of training over three visits, and displaying improved performance during this training.	Tobii X-60 (Tobii)	II
Wang Q et al, 2015	Typically developing children (1-5 y); Adults	The trained looking patterns of adults were altered to be more similar to those evidenced by a normative group of young children. These alterations appear to be retained in post-training sessions and to generalize to presentations of non-trained stimuli.	EyeLink 1000 plus (SR Research)	II
Wang Q et al, 2019	Healthy children, mean age: 37.4 M; children with ASD, mean age: 34 M.	At post-training, the Cue group had higher %Face than the No-Cue group (p = .015). In the No-Cue group %Face decreased Pre- to Post-Training; no decline was observed in the Cue group	Eyelink 1000 Plus (SR Research)	II
Wass S et al, 2011	Typically developing infants. Mean age 339 D	Post-training assessments revealed improvements in cognitive control and sustained attention, reduced saccadic reaction times, and reduced latencies to disengage visual attention. Trend changes were also observed in spontaneous looking behavior during free play, but no change was found in working memory	Tobii 1750 (Tobii)	II
Neurological conditions				
Lévy-Bencheton D et al, 2016	Patients with HVFD after a stroke. Mean age 57 y.	AS training itself proved to induce significant functional improvements in the overall patient group; in the sub-group of adapted patients, and specifically following the adaptation training, an increase of saccade amplitude was observed during the reading task (left-HVFD patients) and the Serial exploration task, and improvement of the visual QoL.	Infrared ET system (Cambridge Research System)	II
Martínez-Moreno JM et al, 2013	NA	NA	Tobii 1750 (Tobii)	NA

Verghese A et al, 2017	HCs, > 18 y	Experiment 1: anti-saccade training led to improvements in the Simon task (i.e., a reduction in the magnitude of the Simon effect) but did not extend to the Stroop task. Experiment 2: training was effective for both the AS and PS groups.	EyeLink 1000 (SR Research)	II

Legend: LoE – level of evidence; Y-years; M- months; D - days NA – not available; HCs – healthy controls;; AS – antisaccade; PS – prosaccade; RS – Rett Syndrome; VP – very preterm; PT – positive training; NT – negative training; BDI - II- Beck depression inventory; IQ – intelligence quotient; ABM – attentional bias modification; AB – attentional bias; AUDIT - alcohol use disorder identification Test ; SPIN - social phobia inventory; ET – eye tracking; ECAT - eye-gaze contingent attention training; ADHD – attention deficit hyperactivity disorder; SWYG – 'Speak With Your Gaze' software; QoL – quality of life; HVFD - homonymous visual field defects.
